# p-Hydroxyphenylpyruvate, an Intermediate of the Phe/Tyr Catabolism, Improves Mitochondrial Oxidative Metabolism under Stressing Conditions and Prolongs Survival in Rats Subjected to Profound Hemorrhagic Shock

**DOI:** 10.1371/journal.pone.0090917

**Published:** 2014-03-05

**Authors:** Antonella Cotoia, Rosella Scrima, Julia V. Gefter, Claudia Piccoli, Gilda Cinnella, Michele Dambrosio, Mitchell P. Fink, Nazzareno Capitanio

**Affiliations:** 1 Department of Medical and Surgical Sciences, University of Foggia, Foggia, Italy; 2 Department of Clinical and Experimental Medicine, University of Foggia, Foggia, Italy; 3 Department of Critical Care Medicine, University of Pittsburgh School of Medicine, Pittsburgh, Pennsylvania, United States of America; 4 Department of Surgery and Anesthesiology, David Geffen School of Medicine at UCLA, Los Angeles, California, United States of America; University of Padova, Italy

## Abstract

The aim of this study was to test the effect of a small volume administration of p-hydroxyphenylpyruvate (pHPP) in a rat model of profound hemorrhagic shock and to assess a possible metabolic mechanism of action of the compound. The results obtained show that hemorrhaged rats treated with 2–4% of the estimated blood volume of pHPP survived significantly longer (p<0.001) than rats treated with vehicle. In vitro analysis on cultured EA.hy 926 cells demonstrated that pHPP improved cell growth rate and promoted cell survival under stressing conditions. Moreover, pHPP stimulated mitochondria-related respiration under ATP-synthesizing conditions and exhibited antioxidant activity toward mitochondria-generated reactive oxygen species. The compound effects reported in the in vitro and in vivo analyses were obtained in the same millimolar concentration range. These data disclose pHPP as an efficient energetic substrates-supplier to the mitochondrial respiratory chain as well as an antioxidant supporting the view that the compound warrants further evaluation as a therapeutic agent.

## Introduction

Trauma death is a leading cause of death throughout the world [1]. The most common cause of death in polytrauma patients is hemorrhagic shock (HS). Hemorrhage leads to reduced tissue perfusion and, if prolonged, irreversible cell damage caused by depletion of substrate availability, [2], [3]. Standard management for HS consists of the intravenous administraton of adequate volumes of sanguinous and asanguinous fluids [4]. However, administration of large volumes of resuscitation fluids can be associated with increased blood loss, exacerbation of acute lung injury and high mortality [5], [6]. Accordingly, investigators have been interested in developing therapeutic approaches, which can prolong survival of patients with HS without requiring the administration of a large volume of resuscitation fluid.

**Figure 1:**
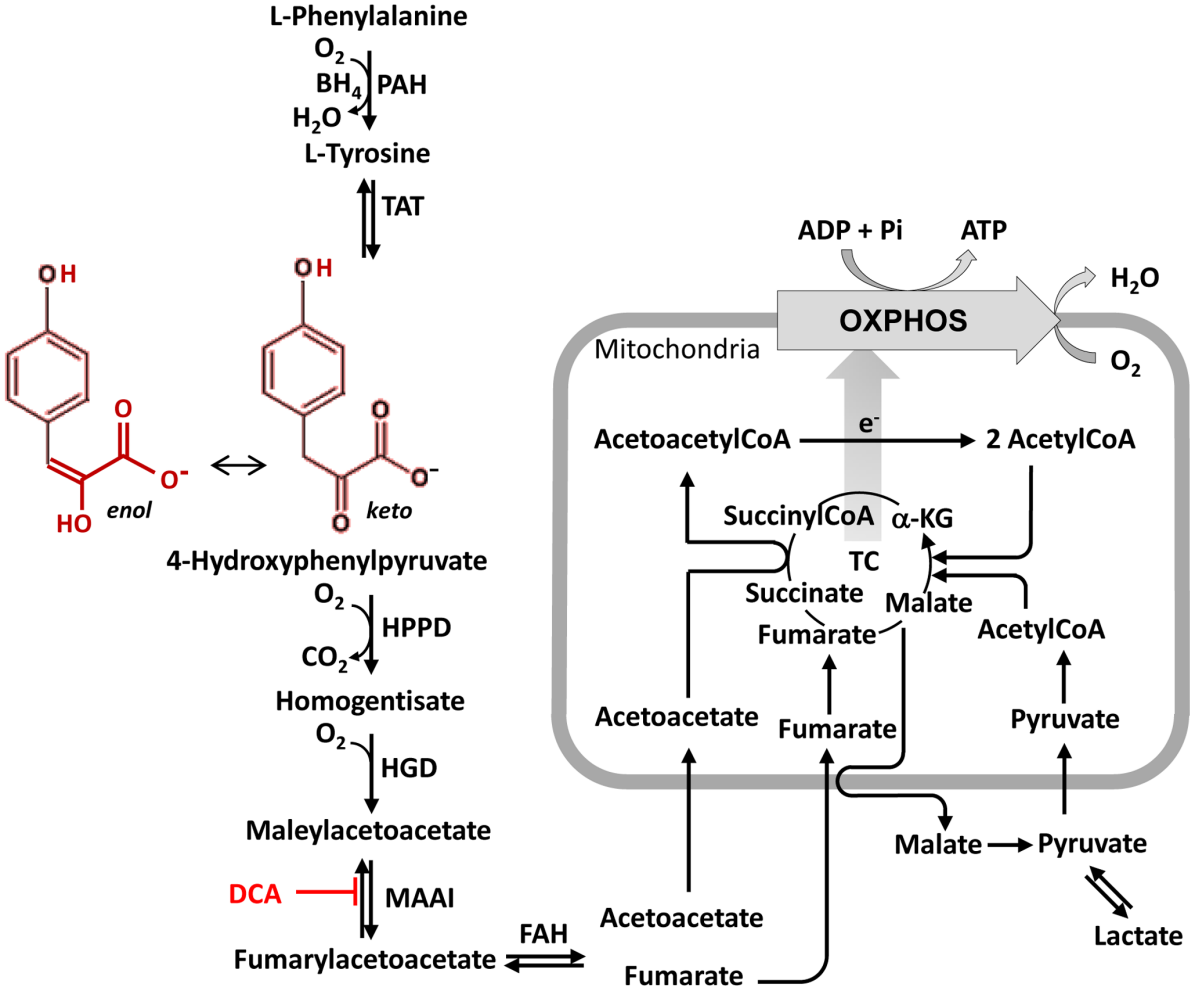
Catabolic pathway of phe/tyr. The scheme illustrates the individual enzymatic steps and intermediates of the catabolism of phe/tyr to acetoacetate and fumarate in the cytoplasmic compartment and their further metabolization within mitochondria. PAH, phenylalanine hydroxylase; BH4, tetrahydrobiopterin; TAT, tyrosine aminotransferase; HPPD, 4-hydroxyphenylpyruvate dioxygenase; HGD, homogentisate dioxygenase; MAAI, maleylacetoacetate isomerase; FAH, fumarylacetoacetate hydrolase; DCA, dichloroacetate; TC, tricaboxylic acid cycle; OXPHOS, oxidative phosphorylation.

Previous work showed that treatment with a resuscitation fluid containing ethyl pyruvate (EP), a hydrolyzable ester of pyruvate, can improve survival in rats or mice subjected to profound HS [7], [8]. However, results from a clinical trial failed to find any evidence for therapeutic effect of EP in patients undergoing cardiac surgery [9]. Nevertheless, many laboratories continue to investigate the therapeutic potential of EP as well as other related compounds [10]–[12]. Para-hydroxyphenylpyruvate (pHPP) is an intermediary metabolite in the catabolic pathway of phenylalanine and tyrosine (Fig. 1) chemically related to EP. In the present study, we found that administration of a small volume of a solution of pHPP can prolong the survival of rats subjected to profound HS. In order to elucidate the mechanism for this protective effect, we carried out additional in vitro studies, using cultured EA.hy 926, human endothelial-like cells unveiling unexpected bio-energizing properties of the compound.

## Materials and Methods

### Cell Cultures

EA.hy 926 human endothelial-derived cells were obtained from American Type Culture Collection (ATCC, Manassas, VA) and maintained at 37°C in DMEM supplemented with 10% fetal bovine serum (FBS) (Hyclone Laboratories, Logan, UT), 1% penicillin/streptomycin, 1% pyruvate, and non-essential amino acid supplement (1% v/v).

### Materials

pHPP, homogentisate (HGA), dichloroacetate (DCA), 2-deoxyglucose (2-DG), rotenone, oligomycin, and carbonyl cyanide 4-(trifluoromethoxy)phenylhydrazone (FCCP) were from Sigma-Aldrich Chemical Co. (St Louis, MO).

### Study Design for Survival of Rats Subjected to Severe Volume-controlled HS

The guidelines for the use of experimental animals of the U.S. National Institutes of Health were followed and approved by the Institutional Animal Care and Use Committee at the University of Pittsburgh. Controlled HS was performed following a previously reported protocol [7]. Briefly, male Sprague-Dawley rats (Charles River Laboratories, Wilmington, MA) weighting 250 to 300 g were anesthetized with intramuscular ketamine HCl at 30 mg/kg and intraperitoneal sodium pentobarbital at 65 mg/kg. Lidocaine (1%, Abbot Laboratories) was provided locally before performing surgical cutdown sites. The right jugular vein was exposed, ligated distally, and cannulated with polyethylene tubing (PE 10) to infuse the test solution. PE 10 was inserted in the isolated and distally ligated femoral artery and attached to a pressure transducer for measurement of the mean arterial pressure (MAP). A silicon catheter (Cronic Cath, Norfolk Medical, Skokie, IL) was introduced into the femoral vein and was used to withdraw blood. Animals were placed in a supine position on a thermal blanket to maintain their body temperature at 37°C. Following surgical preparation and a 5-min stabilization period, rats were subjected to HS. Bleeding was carried out in two phases: 21.7 ml/kg of blood was withdrawn over 20 min, followed immediately by an additional 14 ml/kg over 40 min. The total blood loss was 35.7 ml/kg or approximately 52% of the total estimated blood volume (EBS) [13]. The animals were randomly assigned to one of the following treatment groups (n = 6). *Group 1* was administered 2.8 ml/kg of normal saline (≈4% of EBV); *Group 2* was administered an equivalent volume of 0.25 mmol/kg pHPP solution; *Group 3* received the same 0.25 mmol/kg of pHPP in 50% less of the saline vehicle (i.e., 1.4 ml/kg or ≈2% of EBV); *Group 4* was given 0.25 mmol/kg of homogentisate (HGA) in 2.8 mL/kg of saline; *Group 5* was administered 1 g of dichloroacetate (DCA) in 2.8 mL/kg of saline; *Group 6* received 1 g of DCA per kg in 1.4 mL/kg of saline +0.25 mmol/kg of pHPP in 1.4 mL/kg of saline. The treatment was administered as a continuous infusion during the last 20 min of the hemorrhage period. Blood pressure was monitored continuously as in [7]. Blood-related parameters (i.e. pH, hemoglobin, base excess, pO_2_, pCO_2_, SpO_2_, lactate, and glucose) were determined on 0.3 ml of blood samples, collected through the arterial catheter, at the beginning of hemorrhage (T0) and 30 min after the resuscitation ended (T90) using a commercial blood gas analyzer. Rats were observed until expiration (defined by apnea for >1 min) as direct result of the intervention.

### Impedance Measurements

Impedance-based real time detection of cellular viability was conducted using the xCELLigence system Real-Time Cell Analyzer RTCA-MP (Roche Diagnostics, Penzberg, Germany) in designated 96 well E-plates (Roche, Penzberg, Germany). Recording of cell index values (CI), background subtraction and normalization was performed using the RTCA Software 1.2 (Roche Diagnostics, Penzberg, Germany). The impedance readout as recorded by the xCELLigence system is expressed as arbitrary cell index-values.

### Respirometry

Oxygen consumption rates (OCR) in intact cells were measured by high resolution oxymetry (Oxygraph-2K, Oroboros) in thermostatically controlled tween-chambers (T = 37°C) equipped with a stirring device and a gas-tight plug equipped with a narrow port enabling addition of solutions by microsyringe. Cultured EA.hy 926 cells were detached from the plate by trypsinization, washed in PBS and suspended at 1–2×10^6^ cells/ml in 50 mM KH_2_PO_4_, 10 mM Hepes, 1 mM EDTA, 10 mM glucose, pH 7.4 in both the oxymeter chambers. Respiration of untreated and pHPP-treated cells were assayed in parallel. In the latter case, the assay buffer was supplemented directly with pHPP to obtain the desired final concentration. After achievement of the stationary resting oxygen consumption rate (OCR_R_), 1.0 µg/mL of oligomycin was added (OCR_O_) followed after 5 min by the addition of 75–150 nM of the uncoupler FCCP (OCR_U_); the measured OCRs were corrected for the 2 µM rotenone-insensitive respiration.

### Subcellular Fractionation

Bovine heart mitochondria were isolated by differential centrifugation as described [14]. The supernatatant after the first high-speed centrifugation was taken as the soluble cytoplasmic fraction.

### Laser Scanning Confocal Microscopy Live Cell Imaging of Mitochondrial ROS

Cells cultured at low density on fibronectin-coated 35-mm glass-bottom dishes were incubated for 15 min at 37°C with 0.5 µM MitoSox (Molecular Probes, Eugene, OR) for detection of intra-mitochondrial superoxide (O_2_^⋅−^). Stained cells were examined with a Nikon TE 2000 microscope (images collected using a 60× objective [1.4 NA]) coupled to a Radiance 2100 dual-laser LSCM system (Bio-Rad); red fluorescence was elicited by exciting with the He-Ne laser beam (λ_ex_ 543 nm). Acquisition, storage, and analysis of data were performed with LaserSharp and LaserPix software from Bio-Rad or ImageJ version 1.37 (http://imagej.nih.gov/ij/). Superimposed confocal planes were analyzed by the “stack” function of the LCS-Analysis Tools which produced a xz intensity profile of the average value of the pixels within marked edges, including a single cell, as a function of each focal plane. The integrated value of the xz profile was taken as a measure of the fluorescence intensity of that individual cell relative to the selected emission channel. Correction was made for the minimal background by repeating the procedure in a cell-free field. About one hundred single cells were analyzed for each imaging analysis.

### Statistics

Data are presented as means ± SEM and analyzed using analysis of variance (ANOVA) or two-tailed student t-tests, as appropriate. Survival data were analyzed using Kaplan Meier survival analysis and the log rank test. A value of P<0.05 was considered statistically significant.

## Results

### Effect of pHPP on Survival of Rats Subjected to Experimental HS

**Figure 2:**
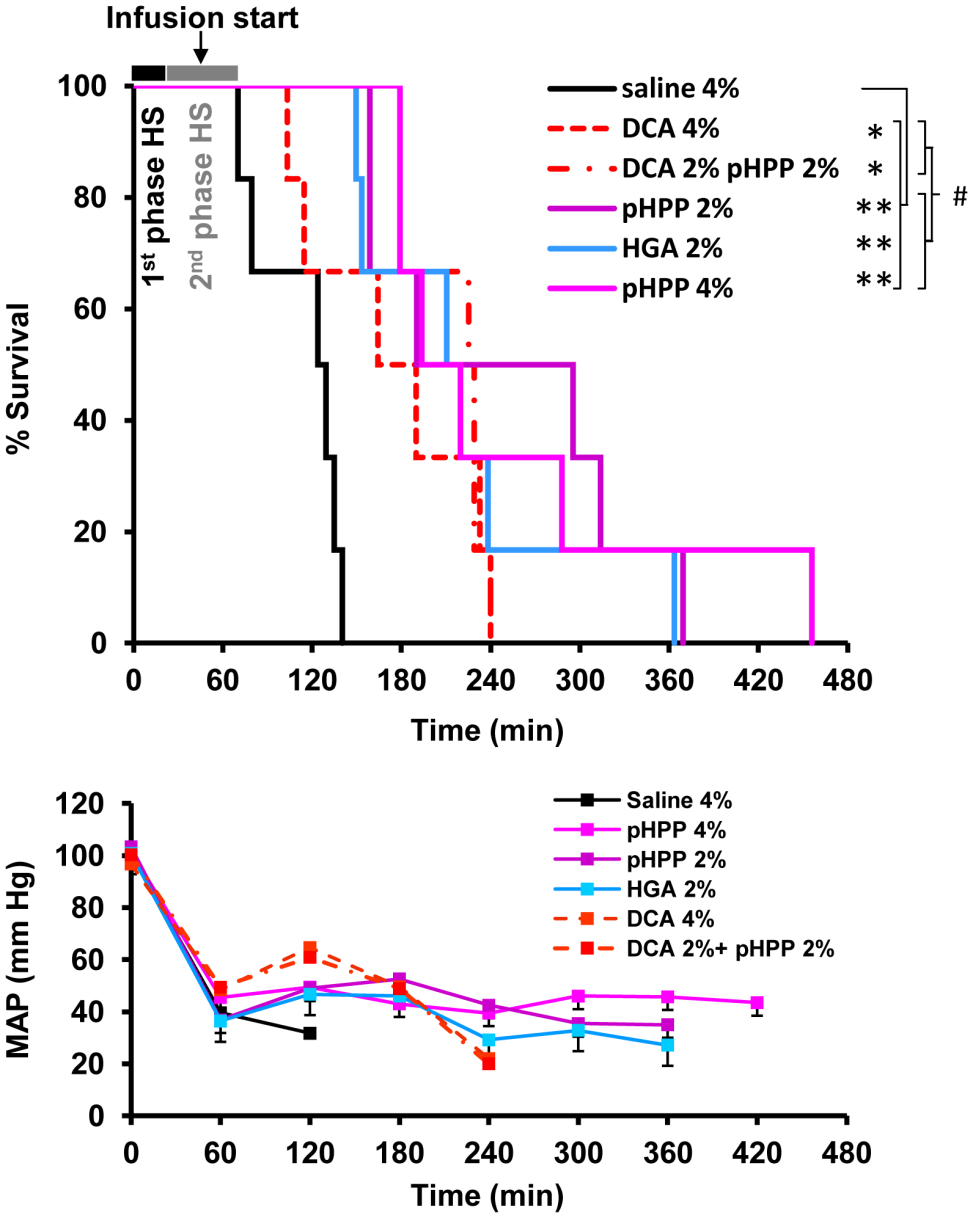
Survival and hemodynamic analysis of rat subjected to severe volume-controlled HS. Rats were subjected to severe volume-controlled HS as described in Materials and Methods. Twenty min before the end of the hemorrhage, rats were infused with either of the following small-volume solutions: saline 2.8 ml/kg (4% of EBV; saline 4%); 0.25 mmol/kg of pHPP in 2.8 ml/kg of saline (4% EBV; pHPP 4%); 0.25 mmol/kg of pHPP in 1.4 ml/kg of saline (2% EBV; pHPP 2%); 0.25 mmol/kg of HGA in 2% EBV of saline (HGA 2%); 1 g/kg of DCA in 4% EBV of saline (DCA 4%); 1 g/kg of DCA in 2% EBV of saline +0.25 mmol/kg of pHPP in 2% EBV of saline (DCA 2%+pHPP 2%). Panel A shows Kaplan-Meier survival curves. * and **^#^**, P<0.05; **, P<0.001. Panel **B** shows mean arterial pressure of rats subjected to HS.

Rats (6 animals per group) were subjected to hemorrhage over a total period of 60 min and the total blood lost was approximately 52% of the EBV (≈18.9 ml/0.275 kg/rat) [13]. Twenty min before the end of the HS, rats were infused with small volumes of normal saline solution without or with added compounds. The infused volumes were ≈4% or ≈2% of the EBV (i.e., the hemorrhaged rats were “resuscitated” with only very small volumes of asanguinous fluids). Figure 2A illustrates the Kaplan-Meyer survival analysis of the experiment. It shows that all animals survived at least 1 h following the initiation of hemorrhage. All animals treated with saline in the control group died within 140 min. The rats treated with 0.25 mmol/kg of pHPP in 4% or 2% of EBV survived significantly longer (p<0.001). Five of 12 animals treated with pHPP survived longer than 4 h and 1 rat survived for 455 min. The infused dose of pHPP resulted in an estimated circulating concentration of about 7 mM.

pHPP, as intermediate of the phenylalanine/tyrosine (phe/tyr) catabolism, is converted to HGA (Fig. 1). Treatment with HGA (2% of EBV) resulted in 33.3% of rats surviving until 5 h. No statistical difference was observed among rats treated with pHPP solution (4% of estimated blood volume), pHPP solution (2% of estimated blood volume), or HGA solution (2% of estimated blood volume).

DCA is an inhibitor of the maleylacetoacetate-*cis*-*trans* isomerase [15], an enzyme, which catalyzes the terminal step in the phe/tyr catabolism pathway. Data shown in Fig. 2A indicate that all rats infused with a millimolar concentrations of either DCA alone or DCA+pHPP died within 4 h.

Rats subjected to HS were continuously monitored for hemodynamic parameters. Before induction of hemorrhage, there were no significant differences in hemodynamic variables and blood gases among groups (not shown). Figure 2B shows that hemorrhage induced a significant decrease in the mean arterial pressure (MAP) during the first 40 min when compared with baseline values. Forty min after the beginning of treatment, at T80 of the experiment, MAP of all groups, except control, yielded a slight increase to values not significantly different among the groups. Thereafter, at T120, MAP was similar in rats treated with pHPP or HGA. Rats treated with DCA or DCA+pHPP tended to have the highest MAP levels (Fig. 2B). No significant changes in arterial blood samples occurred among groups at T0 and T80.

### Effect of pHPP on Cell Growth and Viability

**Figure 3:**
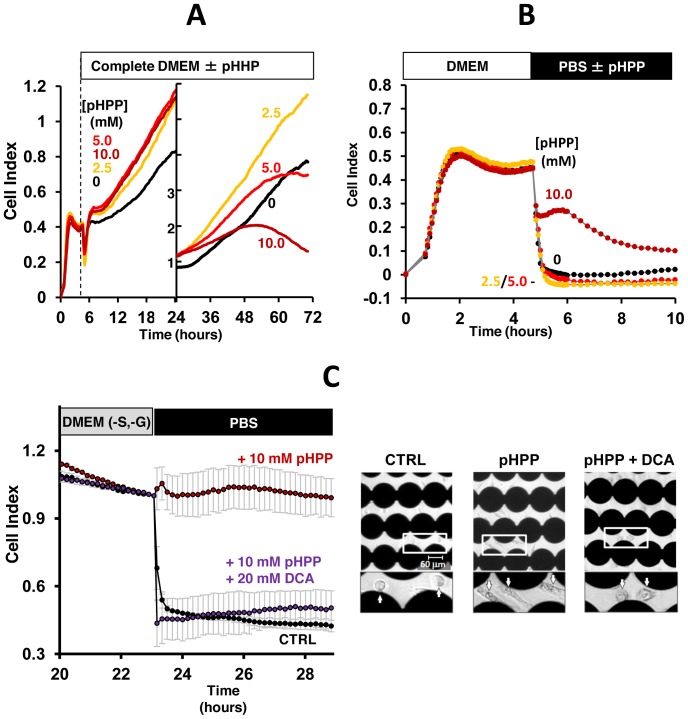
Effect of pHPP on cell growth and cell viability under stressing conditions. EA.hy 926 cells (10^4^ in 100 µl of complete DMEM +10% FBS) were left to adhere to the bottom of the impedentiometric microplate wells in a controlled environment. Impedance was recorded continuously every 5 min and expressed as cell index (CI). (**A**) Once the CI reached a steady value, the medium was substituted with DMEM containing dissolved pHPP at the indicated concentrations and the impedance recorded for three days. The time scale has been split to distinguish early from late phases and the CI scale enlarged in the left part of the diagram. The values shown are means of 2 independent experiments. (**B**) Cells were incubated in complete DMEM for 5 h and then the medium substituted with PBS without or with the indicated concentrations of pHPP and the CI recorded for the following 5 h. The values shown are means of 2 independent experiments. (**C**) Cells were incubated with serum- and glucose-free DMEM (DMEM -S, -G) without or with 10 mM pHPP±20 mM DCA and CI recorded for the following 23 h. Subsequently, the medium was changed to PBS supplemented without or with the same compounds as before. The traces were normalized to the CI values reached before the medium change; each time point is the average ± SEM of 3 independent experiments. The right panel depicts representative phase contrast micrographs of the cells at the end of the experiment; the dark connected circles are the micro-electrodes at the bottom of the impedentiometric wells.

To verify the impact of pHPP on the overall cell physiology, we tested, in vitro, the impact of the compound on the growth rate of the human endothelial-derived EA.hy 926 cells cultured on plates suited for measuring electrical impedance. The impedance measurement provides quantitative information about the biological status of the cells, including cell number, viability, and morphology [16]. Figure 3A shows that supplementation of 2.5–10 mM pHPP to cultured cells in serum-complemented DMEM significantly enhanced the cell index (CI) in the first 24 h. However, in the following 48 h only 2.5 mM pHPP was able to sustain enhanced cell growth.

To assess whether pHPP was an efficient bio-energizer even under conditions associated with cell stress, we tested the effect of the compound on the viability of cells subjected to an abrupt change in the culture medium. As shown in Fig. 3B, substitution of complete DMEM medium with a saline buffer resulted in a dramatic fall of the CI within 30 min. When the saline buffer was supplemented with 10 mM pHPP the decrease of the CI was largely prevented. However, lower concentrations of pHPP (i.e. 2.5 and 5 mM) were, under this condition, inefficient.

Figure 3C shows that when EA.hy 926 cells were preconditioned to promote deficiency of respiratory substrates and serum factors the pro-survival effect of 10 mM pHPP was even stronger when cells were suddenly challenged with the saline buffer. Under this setting, the protective effect of pHPP was completely prevented by DCA.

### Effect of pHPP on Respiratory Activity

**Figure 4:**
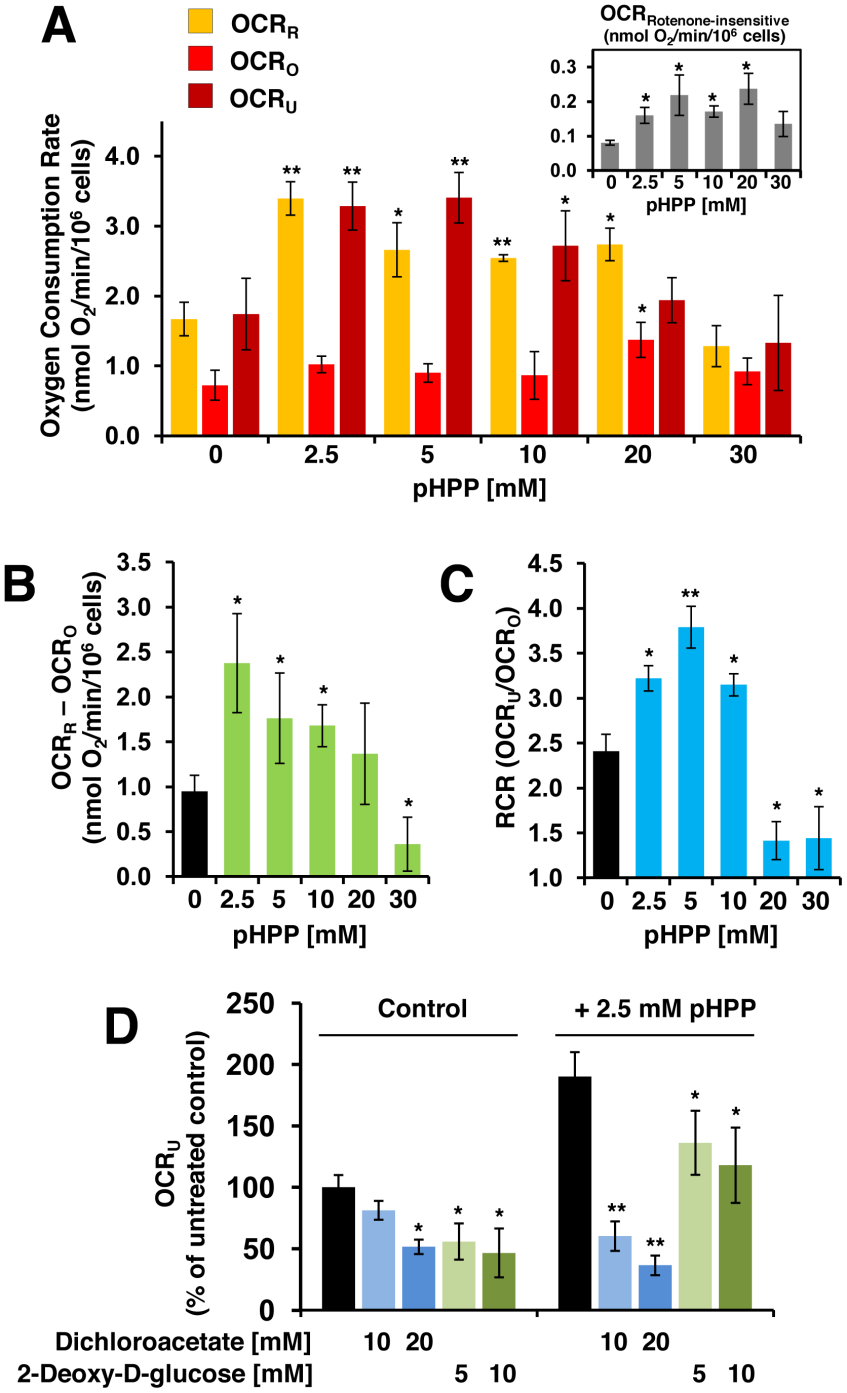
Respirometric analysis of EA.hy 926 cells. Intact EA.hy 926 cells were assayed by high-resolution respirometry in the buffer described in Materials and Methods supplemented with the indicated concentrations of pHPP. Panel **A** depicts OCR measured under resting phosphorylating conditions (OCR_R_), in the presence of FoF1-ATP synthase inhibitor oligomycin (OCR_O_), or in the presence of the protonophoric uncoupler FCCP (OCR_U_). The bar-values were corrected for the residual OCR measured in the presence of the respiratory chain blocker rotenone (shown in the inset) and are means ± SEM of 4–6 independent experiments under each condition. Panel **B** shows OCR linked to ATP synthesis obtained from the values shown in (A) subtracting OCR_O_ from the OCR_R_. Panel **C** shows the respiratory control ratios (RCR) obtained from the values shown in (A) dividing OCR_U_ by the OCR_O_. *, P<0.05; **, P<0.01 (vs untreated). (**D**) OCR_U_ was measured as described in (A); the inhibitors of the tyrosine catabolism and of glycolysis (DCA and 2-DG, respectively) were tested at the indicated concentrations on the respiratory activity assessed in the absence or in the presence of 2.5 mM pHPP. The bar values are percentages of the OCR_U_ measured in control untreated cells and are the means ± SEM of 4–5 independent experiments under each condition. *, P<0.05, **, P<0.01 (vs inhibitor-untreated).

As intermediate in the phe/tyr catabolic pathway pHPP leads to the formation of acetoacetate and fumarate; these compounds are mitochondrial respiratory substrates (Fig. 1). In order to determine whether pHPP is a suitable precursor of respiratory substrates, we tested the effect of pHPP on the respiration of intact EA.hy 926 cells by high resolution oxymetry. Figure 4A shows basal oxygen consumption rates (OCR_R_) of the cells in media supplemented with different concentrations of pHPP. A clear biphasic effect of pHPP is evident. Whereas 2.5 mM pHPP resulted in significant stimulation of oxygen consumption (≈90% increase as compared with untreated cells, P<0.001), higher concentrations of pHPP were associated with an attenuation of the stimulatory effect. When respiratory activity was assayed in the presence of the FoF1-ATP synthase inhibitor, oligomycin, the oxygen consumption rate (OCR_O_) did not change significantly in the tested pHPP concentration range.

Addition of the protonophore FCCP, which uncouples the oxidation of substrates from the phosphorylation of adenosine diphosphate (ADP) recovered the respiratory activity (OCR_U_) to that attained under resting condition and recapitulated a similar dose-response effect of pHPP.

The effect of pHPP on OCR was sensitive to rotenone (or KCN, not shown) and therefore attributable to mitochondrial respiratory chain-dependent respiration. Noticeably, the rotenone-insensitive OCR (i.e., the rate of oxygen consumption that is independent of the mitochondrial respiratory chain) increased significantly in the presence of pHPP (inset of Fig. 4A) leveling off at a 10% of the rotenone-sensitive OCR_R_ measured in the presence of 5 mM pHPP. The phosphorylation-dependent OCR (i.e. OCR_R_-OCR_O_) as well as the respiratory control ratio (i.e. OCR_U_/OCR_O_) displayed a clear and upside-down U-shaped dependence on pHPP concentration (Figs. 4B and 4C).

In order to investigate whether the observed pHPP-mediated increase of respiratory activity was linked to catabolism of the compound, we evaluated the effect of DCA. Figure 4D shows that 10 mM DCA abrogated completely the stimulatory effect on the OCR_U_ attained at 2.5 mM pHPP whereas it caused only a modest decrease of respiratory activity in untreated cells. Conversely, treatment of cells with 2-deoxyglucose (2-DG), an inhibitor of the glycolytic pathway, resulted in a relatively much larger depression of the OCR_U_ in control as compared with the pHPP-treated cells.

**Figure 5:**
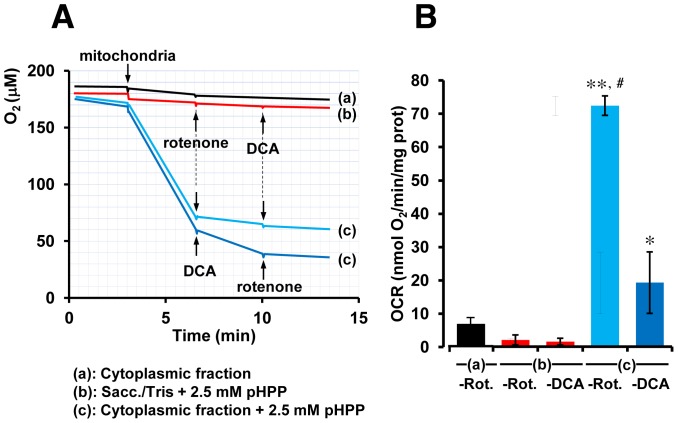
Effect of subcellular fractions on pHPP-dependent respiratory activity. (**A**) Representative respirometric traces. Isolated beef heart mitochondria were added, where indicated, at the concentration of 0.5 mg prot/ml in either of: (trace a) the cytoplasmic fraction isolated from bovine heart homogenate; (trace b) 0.25 M sucrose, 0.1 mM EDTA, 10 mM Tris-HCl pH 7.8 containing 2.5 mM of dissolved pHPP; (traces c) the cytoplasmic subcellular fraction containing 2.5 mM of pHPP. Where indicated, 2 µM rotenone or 10 mM DCA were added. (**B**) The histogram shows the mitochondrial OCR (means ± SEM) of three independent experiments under each condition, measured in the absence (a) or in the presence (b and c) of pHPP; the values shown were corrected for the rotenone (-rot.)- or the DCA (-DCA)-insensitive OCR. * and ** indicate P<0.05 and P<0.001 vs the other conditions; #, P<0.01 ((c)-Rot. vs (c)-DCA).

Finally, since the catabolism of pHPP is expected to occur initially in the cytoplasmic compartment of the cell and then to continue in mitochondria, we examined the effect of pHPP on OCR in subcellular fractions obtained from homogenized bovine heart. Figure 5 shows that the addition of isolated mitochondria to either a medium containing 2.5 mM pHPP or to the soluble cytoplasmic fraction, but without pHPP, did not result in any oxygen consumption. Conversely, when mitochondria were added to the cytoplasmic fraction supplemented with pHPP, a rapid oxygen consumption was observed, which was almost completely inhibited by the mitochondrial respiratory chain inhibitor rotenone and substantially inhibited by 10 mM DCA. We observed a small, but nonetheless detectable OCR, when the cytoplasmic fraction was incubated with pHPP before the addition of isolated mitochondria. This last observation and the mitochondrial-independent oxygen consumption effect shown in Fig. 4A are consistent with the notion that the cytoplasmic conversion of pHPP to acetoacetate and fumarate requires two enzymatic steps catalyzed by O_2_-consuming oxygenases (see Fig. 1).

### Effect of pHPP on Mitochondrial Reactive Oxygen Species Production

**Figure 6:**
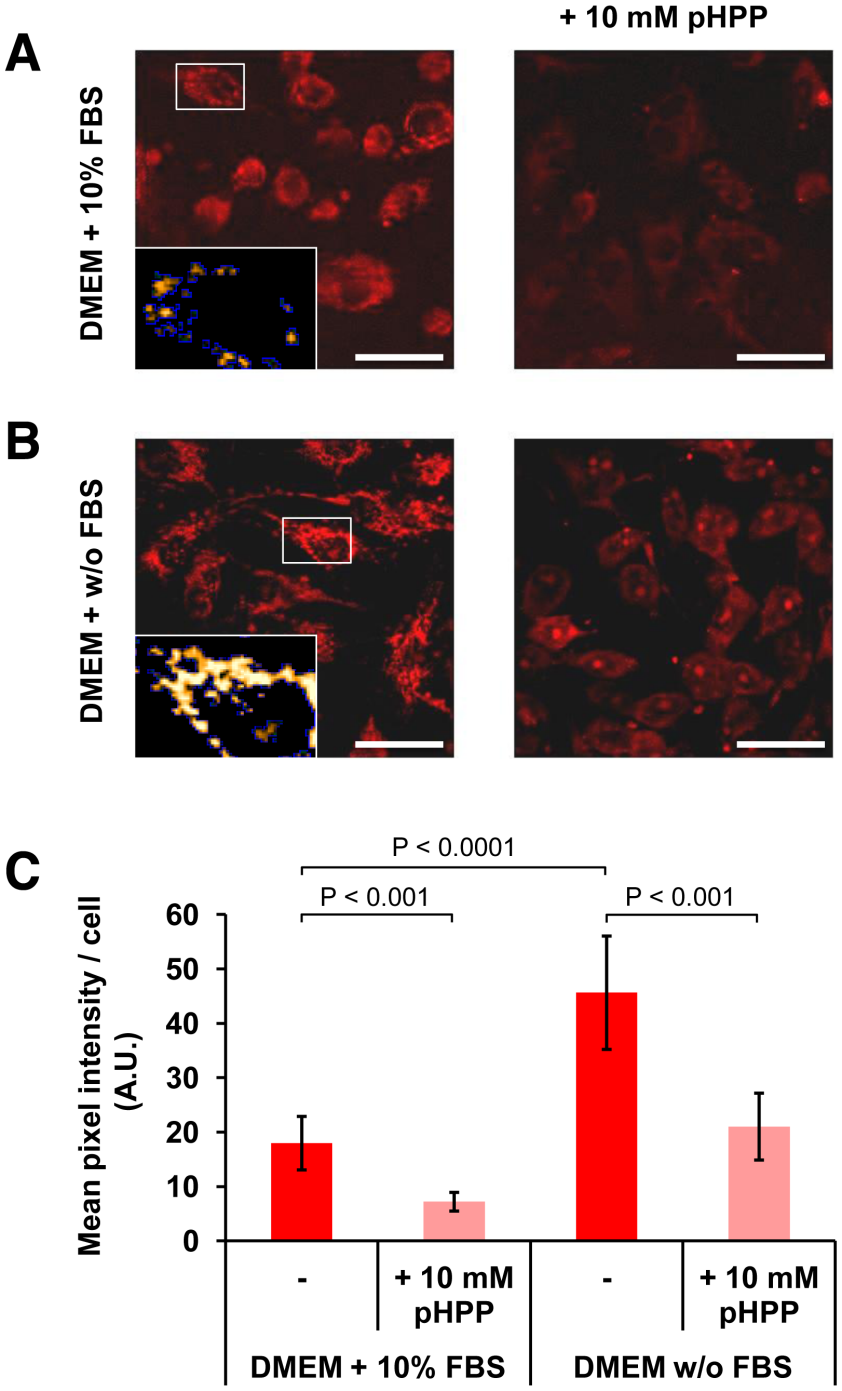
Confocal microscopy analysis of the effect of pHPP on production of mitochondrial superoxide anion in cultured cells. EA.hy 926 cells were grown on coverslips in complete DMEM +10% FBS (**A**) or without FBS (**B**) (for 4 hours) either in the absence and in the presence of 10 mM pHPP followed by addition of the mt-O_2_^.−^ fluorescent probe MitoSox (0.5 µM, 15 min incubation). The pictures are optical fields under each condition as imaged by laser scanning confocal microscopy and are representative of two independent experiments yielding similar outcomes. White bar: 50 µm. The insets show selected enlargements rendered to highlight the intracellular compartmentalization of the fluorescent signal. (**C**) Histogram showing the quantitative analysis of the intracellular fluorescent signal along with the statistical significance of the differences; bars indicate mean values ± SD from 5 randomly selected optical fields/experiment taken under each condition (each optical field contained ≈20 cells). See under Materials and Methods for further details.

Enone containing compounds like EP and pyruvate were shown to function as scavengers of reactive oxygen species (ROS) [17], [19]. To test this property for pHPP, its effect on ROS production by endothelial cells was assessed by confocal microscopy imaging of cells treated with the fluorescent probe MitoSox, which accumulates into mitochondria and reacts preferentially with the superoxide anion (O_2_^⋅−^) therein [20]. Figure 6 shows a representative image of EA.hy 926 cells cultured under normal growth conditions (i.e. DMEM +10% FBS) stained with MitoSox. Basal production of ROS can be appreciated by the probe-related intracellular red fluorescence. To note, the brightest fluorescent signal was punctuate as expected for the mitochondria-targetted probe. Treatment of cells in tween samples with pHPP (10 mM for 45 min) resulted in a clear and significant decrease of the ROS-related fluorescent signal. To further challenge the antioxidant activity of pHPP under pro-oxidative stressing conditions, endothelial cells were growth for 4 hours in the absence of FBS. As shown in Fig. 6 this condition resulted in a significant almost three-fold increase of ROS production (consistent with previous reports [21]), which was largely prevented by pHPP and kept within the basal levels.

Collectively these data suggest that pHPP exerts a cellular protective effect not only providing substrate for fuel but also via an antioxidant mechanism. This latter might be accomplished either directly by ROS-scavenging chemical properties or indirectly providing antioxidant metabolites.

## Discussion

Although the mechanism of organ failure and dysfunction after HS and re-perfusion has not been completely elucidated, inadequate tissue perfusion leads to gross metabolic disturbance, mitochondria dysfunction/energy failure, severe acidosis, and the generation of ROS, leading to consumption of endogenous antioxidants [22], [23]. However, rapid replacement with large-volume resuscitation through the use of intravenous fluids and blood products [4] has been shown to increase hemorrhage volume and decrease survival compared with either low-volume or delayed fluid replacement [24]. Therefore, the potential for resuscitating injured patients with HS without the need for blood and blood product remains quite attractive [25].

In the present study, we tested the *in vivo* pro-survival effects of pHPP in a rat model of profound HS. The protocol was designed to closely mimic the preclinical management of poly-trauma patients. The results shown in Fig. 2 show that survival time was significantly prolonged when hemorrhaged rats were treated with a small volume of pHPP solution in the absence of major sanguinous or asanguinous resuscitation. In a previous study, Macias *et al.* [26] showed that hemorrhaged rats treated with 0.5 mmol/kg of EP dissolved in 2.8 mL/kg of saline survived significantly longer compared with rats treated with the same volume of vehicle alone. Herein, we showed that an even smaller molar dose of pHPP (0.25 mmol/kg) in the same volume of saline (2.8 mL/kg) conferred a survival advantage, and the prolongation of survival was greater than the one previously reported by Macias and colleagues in their study of EP solution. Remarkably, we found that the same amount of pHPP given in 2% EBV of saline resulted in approximately 50% survival at 4 h after rats were subjected to more than 50% blood loss. To note, the estimated concentrations of pHPP in the residual blood volume after hemorrhage were 6.7 mM and 7 mM following the 4% and 2% of the EBV perfusion, respectively. Furthermore, it is important to note that treatment with pHPP solution prolonged survival without causing a substantial increase in blood pressure. Thus, treatment with pHPP could be used to “buy time” until definitive control of surgical bleeding without promoting excessive further blood loss [27].

Notably, there was no statistically significant difference between HGA (in 2% EBV) and pHPP (either in 4% or 2% of EBV). This finding supports the notion that metabolism of pHPP via the phe/tyr catabolic pathway is important for its therapeutic effect. Consistently, when DCA was co-administered with pHPP or infused alone, only 1 of 6 animals in each group survived until 4 h.

Cellular ATP levels are depleted in rats (and, presumably, humans) with shock due to hemorrhage [28]. Interestingly, no significant benefit with regard to tissue bioenergetics was observed following resuscitation with ethyl pyruvate-supplemented Ringer’s solution in a swine model of HS [29]. Although we did not measure cellular ATP levels in the rat HS experiments reported here, we clearly demonstrated that pHPP effectively supports mitochondrial respiration in cultured EA.hy 926 cells. The relatively high rate of pHPP oxidative degradation is likely due to the fact that pHPP bypasses tyrosine transaminase and the phenylalanine hydroxylase steps in the phe/tyr catabolic pathway; both of these enzymatic reactions are tightly regulated and limit the rate of catabolism of these amino acids [30], [31].

The end-products of pHPP catabolism in the cytoplasm are fumarate and acetoacetate. Both of these compounds can enter into mitochondria and support the tricarboxylic acid cycle either as intermediates or substrates (see scheme in Fig. 1 and experimental evidences provided in Fig. 5). Importantly, the enhanced delivery of reducing equivalents to the mitochondrial respiratory chain is fully coupled to the synthesis of ATP as shown by both the enhanced oligomycin-sensitive OCR and RCR (Fig. 4). Consequently, pHPP appears to be an efficient bio-energizer providing extra ATP to support adaptation to stressing conditions (over a time scale of a few minutes) or to foster normal cell growth (over a time scale measured in days) as shown in Fig. 3. This property would result beneficial to cells/tissues experiencing a shortage of metabolic substrates, serum factors or oxygenation as that attained following a severe HS. Most notably, the in vitro-tested concentrations of pHPP were in the same millimolar range used in the in vivo model of HS. This suggests that, at least in part, the therapeutical efficacy of pHPP can be traced back to its metabolic activity.

A further property that we tested and proved for pHPP was its antioxidant activity (Fig. 6). The enone moiety present in the molecule is likely to provide the chemical basis for its ROS scavenging action as proposed for EP and pyruvate sharing a similar feature [18]. It is worth noting that since the ROS-probe used detects intramitochondrial ROS this implies that pHPP (or related metabolites) exerts its antioxidant action within the mitochondrial compartment. Whatever is the precise mechanism, which warrants further investigation, the antioxidant activity of pHPP may contribute to counter-act the cytotoxic effect of a pro-oxidant conditions like that establishing under hemorrhagic shock. Other possible activities of pHPP (anti-inflammatory in particular) are currently under scrutiny.

In conclusion, the data presented support the view that pHPP might be useful for the management of traumatically injured patients in order to prolong their period of survival time after losing large quantities of blood. Additional pre-clinical studies with this compound appear to be warranted.

## References

[1] Kauvar DS, Lefering R, Wade CE (2006) Impact of hemorrhage on trauma outcome: an overview of epidemiology, clinical presentations, and therapeutic considerations. J Trauma 60(6 Suppl): S3–11. 10.1097/01.ta.0000199961.02677.19 16763478

[2] Peitzman AB, Billiar TR, Harbrecht BG, Kelly E, Udekwu AO, et al (1995) Hemorrhagic shock. Curr Probl Surg 32: 925–1002. 7587344 10.1016/s0011-3840(05)80008-5

[3] Cairns CB (2001) Rude unhinging of the machinery of life: metabolic approaches to hemorrhagic shock. Opin Crit Care 7: 437–443. 10.1097/00075198-200112000-00011 11805547

[4] McSwain NE, Champion HR, Fabian TC, Hoyt DB, Wade CE, et al.. (2010) State of the art of fluid resuscitation 2010: prehospital and immediate transition to the hospital. J Trauma 70(5 Suppl): S2–10. 10.1097/TA.0b013e31821b201d 21841563

[5] Riddez L, Drobin D, Sjostrand F, Svensen C, Hahn RG (2002) Lower dose of hypertonic saline dextran reduces the risk of lethal rebleeding in uncontrolled hemorrhage. Shock 17: 377–382. 12022757 10.1097/00024382-200205000-00006

[6] Shah KJ, Chiu WC, Scalea TM, Carlson DE (2002) Detrimental effects of rapid fluid resuscitation on hepatocellular function and survival after hemorrhagic shock. Shock 18: 242–247. 12353925 10.1097/00024382-200209000-00007

[7] Tawadrous ZS, Delude RL, Fink MP (2002) Resuscitation from hemorrhagic shock with Ringer’s ethyl pyruvate solution improves survival and ameliorates intestinal mucosal hyperpermeability in rats. Shock 17: 473–477. 12069183 10.1097/00024382-200206000-00006

[8] Yang R, Gallo DJ, Baust JJ, Watkins SK, Delude RL, et al (2002) Effect of hemorrhagic shock on gut barrier function and expression of stress-related genes in normal and gnotobiotic mice. Am J Physiol Regul Integr Comp Physiol 283: R1263–1274. 12376421 10.1152/ajpregu.00278.2002

[9] Bennett-Guerrero E, Swaminathan M, Grigore AM, Roach GW, Aberle LG, et al (2009) A Phase II Multicenter Double-Blind Placebo-Controlled Study of Ethyl Pyruvate in High-Risk Patients Undergoing Cardiac Surgery With Cardiopulmonary Bypass. J Cardiothorac Vasc Anesth 23: 324–329. 18835526 10.1053/j.jvca.2008.08.005

[10] Leelahavanichkul A, Yasuda H, Doi K, Hu X, Zhou H, et al (2008) Methyl-2-acetamidoacrylate, an ethyl pyruvate analog, decreases sepsis-induced acute kidney injury in mice. Am J Physiol Renal Physiol 295: F1825–35. 18922884 10.1152/ajprenal.90442.2008 PMC2604833

[11] Sappington PL Cruz RJ Jr, Harada T, Yang R, Han Y, et al (2005) The ethyl pyruvate analogues, diethyl oxaloproprionate, 2-acetamidoacrylate, and methyl-2-acetamidoacrylate, exhibit anti-inflammatory properties in vivo and/or in vitro. Biochem Pharmacol 70: 1579–1592. 16226725 10.1016/j.bcp.2005.08.015

[12] Kim SW, Kim HJ, Shin JH, Kim ID, Lee JE, et al (2011) Robust protective effects of a novel multimodal neuroprotectant oxopropanoyloxy benzoic acid (a salicylic acid/pyruvate ester) in the postischemic brain. Mol Pharmacol 79: 220–228. 21036874 10.1124/mol.110.067520

[13] Probst RJ, Lim JM, Bird DN, Pole GL, Sato AK, et al (2006) Gender differences in the blood volume of conscious Sprague-Dawley rats. J Am Assoc Lab Anim Sc. 45: 49–52. PMC1409819 16542044

[14] Graham JM (2001) Isolation of mitochondria from tissues and cells by differential centrifugation. Curr Protoc Cell Biol Chapter 3: Unit 3.3. 10.1002/0471143030.cb0303s04 18228355

[15] Lantum HB, Cornejo J, Pierce RH, Anders MW (2003) Perturbation of maleylacetoacetic acid metabolism in rats with dichloroacetic Acid-induced glutathione transferase zeta deficiency. Toxicol Sci 74: 192–202. 12730618 10.1093/toxsci/kfg104

[16] Ke N, Wang X, Xu X, Abassi YA (2011) The xCELLigence system for real-time and label-free monitoring of cell viability. Methods Mol Biol 740: 33–43. 21468966 10.1007/978-1-61779-108-6_6

[17] Varma SD, Devamanoharan PS, Ali AH (1998) Prevention of intracellular oxidative stress to lens by pyruvate and its ester. Free Rad Res 28: 131–135. 10.3109/10715769809065799 9645390

[18] Kao KK, Fink MP (2010) The biochemical basis for the anti-inflammatory and cytoprotective actions of ethyl pyruvate and related compounds. Biochem Pharmacol 80: 151–159. 20230800 10.1016/j.bcp.2010.03.007

[19] Famili A, Ammar DA, Kahook MY (2013) Ethyl pyruvate treatment mitigates oxidative stress damage in cultured trabecular meshwork cells. Mol Vis 19: 1304–1309. 23805037 PMC3692399

[20] Robinson KM, Janes MS, Pehar M, Monette JS, Ross MF, et al (2006) Selective fluorescent imaging of superoxide in vivo using ethidium-based probes. Proc Natl Acad Sci U S A 103: 15038–43. 17015830 10.1073/pnas.0601945103 PMC1586181

[21] Kuznetsov AV, Kehrer I, Kozlov AV, Haller M, Redl H, et al (2011) Mitochondrial ROS production under cellular stress: comparison of different detection methods. Anal Bioanal Chem 400: 2383–2390. 21336935 10.1007/s00216-011-4764-2

[22] Macias CA, Chiao JW, Xiao J, Arora DS, Tyurina YY, et al (2007) Treatment with a novel hemigramicidin-TEMPO conjugate prolongs survival in a rat model of lethal hemorrhagic shock. Ann Surg 245: 305–314. 17245186 10.1097/01.sla.0000236626.57752.8e PMC1876982

[23] Fink MP, Macias CA, Xiao J, Tyurina YY, Delude RL, et al.. (2007) Hemigramicidin-TEMPO conjugates: novel mitochondria-targeted antioxidants. Crit Care Med 35(9 Suppl): S461–7. 10.1097/01.CCM.0000279192.96303.E7 17713394

[24] Dubick MA, Atkins JL (2003) Small-volume fluid resuscitation for the far-forward combat environment: current concepts. J Trauma 54(5 Suppl): S43–45. 10.1097/01.TA.0000064514.42470.3B 12768102

[25] Cotton BA (2011) Alternative fluids for prehospital resuscitation: “pharmacological” resuscitation fluids. J Trauma 70(5 Suppl): S30–31. 10.1097/TA.0b013e31821a55af 21841566

[26] Macias CA, Chiao JW, Harada T, Fink MP (2005) Small volume resuscitation with a solution containing ethyl pyruvate improves survival in a lethal model of hemorrhagic shock. Crit Care Med 33: A32.

[27] Owens TM, Watson WC, Prough DS, Uchida T, Kramer GC (1995) Limiting initial resuscitation of uncontrolled hemorrhage reduces internal bleeding and subsequent volume requirements. J Trauma 39: 200–7 discussion 208–9. 7674386 10.1097/00005373-199508000-00004

[28] Taylor JH, Beilman GJ, Conroy MJ, Mulier KE, Myers D, et al (2004) Tissue energetics as measured by nuclear magnetic resonance spectroscopy during hemorrhagic shock. Shock 21: 58–64. 14676685 10.1097/01.shk.0000101674.49265.93

[29] Mulier KE, Beilman GJ, Conroy MJ, Taylor JH, Skarda DE, et al (2005) Ringer’s ethyl pyruvate in hemorrhagic shock and resuscitation does not improve early hemodynamics or tissue energetics. Shock 23: 248–252. 15718923

[30] Dickson AJ, Marston FA, Pogson CI (1981) Tyrosine aminotransferase as the rate-limiting step for tyrosine catabolism in isolated rat liver cells. FEBS Lett 127: 28–32. 6113991 10.1016/0014-5793(81)80333-x

[31] Kaufman S (1999) A model of human phenylalanine metabolism in normal subjects and in phenylketonuric patients. Proc Natl Acad Sci U S A 96: 3160–3164. 10077654 10.1073/pnas.96.6.3160 PMC15912

